# Knowledge-based analysis of microarrays for the discovery of transcriptional regulation relationships

**DOI:** 10.1186/1471-2105-11-S1-S8

**Published:** 2010-01-18

**Authors:** Junhee Seok, Amit Kaushal, Ronald W Davis, Wenzhong Xiao

**Affiliations:** 1 Stanford Genome Technology Center, 955 California Avenue, Palo Alto, California, 94305, USA; 2 Department of Electrical Engineering, Stanford University, 350 Serra Mall, Stanford, California, 94305, USA; 3 Massachusetts General Hospital, Harvard Medical School, 55 Fruit Street, Boston, Massachusetts, 02114, USA

## Abstract

**Background:**

The large amount of high-throughput genomic data has facilitated the discovery of the regulatory relationships between transcription factors and their target genes. While early methods for discovery of transcriptional regulation relationships from microarray data often focused on the high-throughput experimental data alone, more recent approaches have explored the integration of external knowledge bases of gene interactions.

**Results:**

In this work, we develop an algorithm that provides improved performance in the prediction of transcriptional regulatory relationships by supplementing the analysis of microarray data with a new method of integrating information from an existing knowledge base. Using a well-known dataset of yeast microarrays and the Yeast Proteome Database, a comprehensive collection of known information of yeast genes, we show that knowledge-based predictions demonstrate better sensitivity and specificity in inferring new transcriptional interactions than predictions from microarray data alone. We also show that comprehensive, direct and high-quality knowledge bases provide better prediction performance. Comparison of our results with ChIP-chip data and growth fitness data suggests that our predicted genome-wide regulatory pairs in yeast are reasonable candidates for follow-up biological verification.

**Conclusion:**

High quality, comprehensive, and direct knowledge bases, when combined with appropriate bioinformatic algorithms, can significantly improve the discovery of gene regulatory relationships from high throughput gene expression data.

## Background

The rapid accumulation of high-throughput genomic data provides the foundation to computationally infer previously unknown regulatory relationships between transcription factors and their target genes. DNA microarrays have been widely applied to measure genome-wide mRNA abundance of many organisms under different conditions. To infer regulatory relationships from gene expression data, many of the computational analysis methods developed to date focus only on the experimental dataset itself, by evaluating similarities between expression patterns using either clustering algorithms such as hierarchical clustering [1] and self-organizing maps [2], probabilistic graphical models [3], or the context likelihood of relatedness (CLR) [4].

Most of these methods do not take full advantage of the vast amounts of molecular interaction information accumulated by the research community, including gene-gene regulatory interactions [5,6], DNA-protein binding [7], metabolic pathways [8] as well as sets of genes related to diseases or cell functions [9]. Knowledge bases collect, curate, and perform quality control of the previous findings, either manually by expert biologists [5] or computationally by artificial intelligence programs [6], from peer-reviewed publications [5,6] and/or from data of high-throughput experiments [7]. Different knowledge bases can vary in many aspects, such as the source of the original information, quality of the data curation, and the comprehensiveness of the database. Recent approaches have begun to utilize prior knowledge for a variety of computational analyses of genomic data including metabolic network modeling [10], inference of activities of transcription factors [11], significance analysis and classification using gene sets [12-15], and inference of transcriptional regulation relationships [16-18]. These studies demonstrate that knowledge-based methods can help uncover important biological information from high-throughput genomic data. We have developed an improved algorithm to systematically utilize information in a large collection of previously known interactions between transcription factors (TFs) and target genes (TGs) for the discovery of previously unknown gene regulation. First, we estimated the regulatory signal of a TF, mainly from the centroid expression of the known co-regulated TGs of the TF. We then built a predictor to discover new regulatory pairs based on the relationship between the regulatory signal of the TF and the expression of the candidate genes. We also used our approach to characterize the various properties of knowledge bases and investigated their performance in regulatory-pair prediction.

Using the Yeast Proteome Database (YPD), a large database created from manual curation of peer-reviewed publications by experts [5], we analyzed the utility of the knowledge base in inferring new transcriptional regulations from gene expression data sets of *Saccharomyces cerevisiae*. We show that the knowledge base provides valuable additional information to characterize pairs of genes linked by transcriptional regulations, and predictions made using prior knowledge have significantly better performance than those from experimental data alone. Further, we tested prediction performance using knowledge bases of different characteristics, and show that a knowledge base with comprehensive, direct and high-quality interactions results in better performance in analysis. Finally, we predicted 547 new regulatory pairs through genome-wide analysis, and by evaluating these pairs in a ChIP-chip binding dataset [7] and a growth fitness data set [19], we suggest that they can be reasonable candidates for further biological verification.

## Methods

### Data sources

We evaluated the usefulness of a knowledge base for finding new regulatory pairs on a well-known collection of 643 microarrays of yeast, described by Stuart *et al*. [20]. We include in our study the 5,940 genes which have missing data rates of less than 30%; the overall missing data rate in the resulting dataset is less than 1%.

We describe knowledge bases containing information manually curated from literature as having "direct information", and those with information extracted from high-throughput data as having "indirect information". Here we assume that findings of a TF and its TGs specifically reported in peer-reviewed publications are more likely to be direct and specific regulations. In addition, we define comprehensiveness of a knowledge base to be proportional to the number of regulatory pairs in the collection. Quality of a knowledge base is quantified as the number of false positive interactions for a given number of true gene interactions. The repository of gene interaction pairs of transcriptional regulation from the YPD is an example of a direct knowledge base. We extracted 3,043 pairs of transcriptional interactions between 523 TFs and 919 TGs from the YPD for inclusion in our analysis of this direct knowledge base.

To evaluate the validity of our genome-wide predicted regulatory relations, ChIP-chip binding data from Harbison *et al*. [7] and a growth fitness dataset of homozygous gene deletions having growth fitness data of 3,961 genes in 418 conditions [19] were used as independent data sources. The growth fitness score was calculated as the log ratio of the growth rate of the gene deletion to that of the control.

### Representation of gene expression

Let *E*_*ij *_be the expression level for gene *i *of array *j*, and *n *be the total number of the arrays in our collection. The *naïve-representation *(NR) for gene *i *is defined as the following.(1)

With a knowledge base, we can use the known co-regulated TGs to define a new representation. Let *S*_*i *_be the set of TGs regulated by gene *i *in the knowledge-base. We define the *knowledge-based-representation *(KBR) for gene *i *as follows:(2)

*N*_*i *_is the number of TGs regulated by the gene *i *in the knowledge base. The KBR represents the centroid expression of gene *i *and TGs that it regulates. If a gene is not a TF or there is no known TG for the gene, the KBR of the gene is equal to the NR. The KBR of a TF can also be considered as reflecting the regulatory signal for the TF.

### Classification methods and generation of negative sets

To evaluate the performance of prediction with knowledge-based analysis, we used two classification algorithms, correlation cutoff and support vector machine (SVM). The correlation cutoff method measures the correlation between representations of a TF and a candidate TG. Pairs with a correlation value greater than a certain cutoff are called true regulatory pairs. A Pearson correlation coefficient was used as a measurement of correlation. The correlation approach can be applied to both the NR and KBR for a pair of genes. The NR cutoff method uses the correlation between NRs of a TF and a TG, while the KBR cutoff method uses the correlation between KBRs. Receiver-operation-characteristic (ROC) curves of cutoff methods are plotted by changing cutoff thresholds. SVM uses an optimal marginal classification, which builds the decision boundary maximizing distance from the boundary to the training samples of each class in the feature space [21]. It has been shown to have good scalability to large data sets with high accuracy [22]. We can use NR and KBR to build a feature for SVM. The NR SVM method builds a feature vector of a regulatory pair by concatenating the NRs of the TF and the TG. The procedure for the KBR SVM approach is identical except for the use of KBRs instead of NRs. The NR SVM method is the same as used in Qian *et al*. [17]. ROC curves of SVM methods are plotted by changing thresholds of prediction scores. The prediction score of an SVM is defined as the distance of a tested sample from the optimal decision boundary. Here, we use SVM-light [23], an open-source implementation of the SVM algorithm, with a radial kernel function.

In order to build and evaluate predictors of cutoff and SVM methods, the 3,043 pairs of transcriptional interactions extracted from YPD served as the positive regulatory set, and the negative training and test sets in which the pairs have no regulatory interactions were randomly selected from all possible TF-TG pairs under the assumption that genome-wide regulatory relations are sparse [16,17]. In order to reflect sparseness of true regulatory relations, we chose negative sets ten times larger than positive sets, as in Qian *et al*. [17]. In addition, negative sets were generated using the distribution of positive sets so that the number of false relations of a TF would be proportional to the number of true relations of the TF.

### The CLR and SEREND algorithm

Software implementations of the CLR and SEREND algorithm were obtained from their supporting websites [4,18]. The algorithms were applied to our yeast data with default parameters. The SEREND algorithm was performed without sequence motifs information for a fair comparison, and the expression classifier scores were used as the final prediction scores.

## Results and discussion

### Representation of gene expression using a knowledge base

There have been many studies to infer new gene regulations based on gene expression data alone. These methods are based on examining the correlation in gene expression pattern between a TF and a candidate TG under the assumption that a TF and its regulated TGs will be co-expressed and have higher correlation or richer mutual information in expression [3,4]. Although this model may fit reasonably well for a subset of known TFs [3], we expect that it does not hold for many others. Many TFs require complex post-translational modifications to switch on the transcription of TGs; this activation process is often independent of the expression level of the TF in the absence of a self regulatory loop. Therefore, expression of a TF does not necessarily imply activation and the transcription of its TGs.

**Figure 1:**
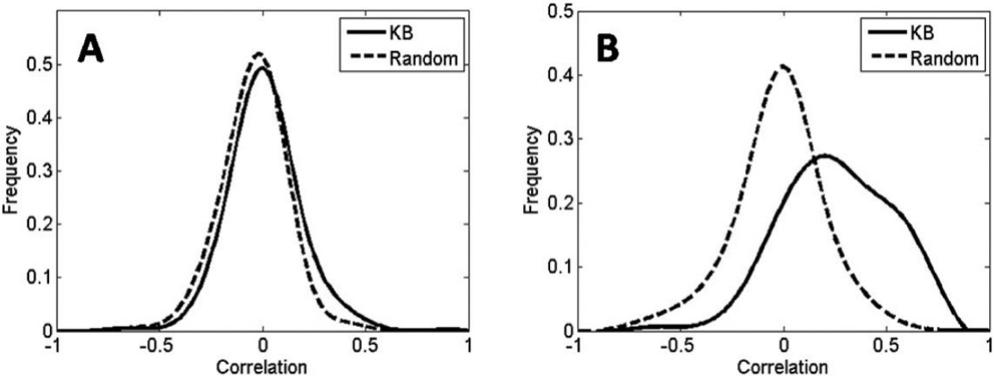
**Distributions of correlation coefficients**. **(A) **Correlations of gene expressions between TFs and their TGs. **(B) **Correlations between gene expressions of TGs and the centroids of other TGs co-regulated by the same TFs in the knowledge base.

To illustrate the challenge of inferring regulatory relations with only co-expression patterns, we measure correlation coefficients between expression levels of a TF and its TG in a well-known collection of 643 microarrays of yeast [20]. For the positive regulatory set, we collected a set of 3,043 known regulatory pairs from the YPD database, while the negative regulatory sets were randomly selected from all possible TF-TG pairs minus the 3,043 positive pairs as described in Methods. As shown in Figure 1A, the expression profile of a single TG did not correlate well with its TF for the true positive regulatory pairs from the knowledge base. Comparing with the random negative pairs, the distribution of correlation coefficients of the true regulatory pairs showed a similar Gaussian-like curve and peak position, despite of small shift. Although this is a naïve measurement, it clearly shows the difficulty to distinguish true and false regulatory relations from gene expression data alone.

The knowledge base of gene regulations provides valuable additional information. For each of the known TFs, there is a set of confidently identified TGs that the TF regulates. We expect that TGs regulated by the same TF are likely to share similar expression profiles. To verify this, we measured correlation coefficients over the same data set as in Figure 1A. For the true regulation set from the knowledge base, a correlation coefficient is calculated between the expression level of a TG and the centroid of expression levels of the other TGs regulated by the same TF. In contrast, for the random regulatory pairs, a correlation coefficient is calculated between a gene and the centroid of the known set of TGs regulated by a randomly chosen TF. These two distributions were distinct - the distribution of the true regulatory pairs exhibited higher correlation values compared to the random false pairs (Figure 1B). Therefore prior knowledge of regulatory relationships provides us unique characteristics of true regulatory pairs that are distinguishable from the false pairs. Here, to reduce the noise of individual genes, we chose to use the correlation between the centroid of the set of known co-regulated genes and a candidate gene instead of individual gene correlations.

### Improvements in predicting transcriptional regulations using knowledge-based analysis

**Figure 2:**
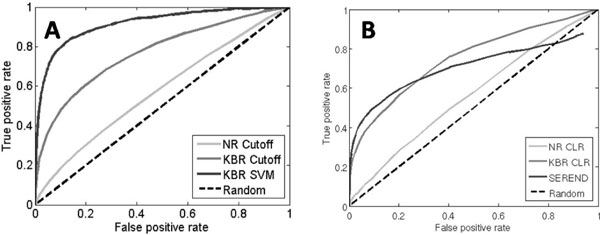
**2-fold cross-validation performances of prediction methods**. **(A) **ROC curves of the regulatory predictions using different methods with a knowledge base (KBR cutoff and KBR SVM) and without a knowledge base(NR cutoff). **(B) **ROC curves of the CLR algorithms with and without prior knowledge, and the expression classifier of the SEREND algorithm.

Next, we compare methods using either naïve-representation (NR) with the microarray data alone or knowledge-based-representation (KBR) that includes both the microarray data and the collection of prior knowledge, on the same gene expression data set as the previous section. The KBR was designed to include both TG expression terms which are expected to be dominant for TFs with many known TGs, and TF expression which can be informative for TFs with few or no known TGs. The true and random regulatory sets in the previous section were used as the positive and negative sets respectively, and two-fold cross-validations was performed for all TFs together. For the cross-validation, training and test sets were randomly separated and mutually exclusive, and the KBRs were encoded using information from training sets only. To evaluate the impact of the knowledge base driven analysis, we first used a simple correlation cutoff classifier; that is, if the correlation between a representation of a TF and a candidate TG was larger than a certain cutoff value, the regulatory pair was labeled a true regulation, and otherwise it was labeled false. Predictions with a knowledge base show better performance than without a knowledge base (Figure 2A). At a 10% false positive rate, the NR cutoff (prediction without a knowledge base) corresponded to a 18% true positive rate while the KBR cutoff (prediction with a knowledge base) had a 48% true positive rate. In addition, a SVM method utilizing known regulations in a knowledge base to predict new regulations shows the best performance, a 80% true positive rate at a 10% false positive rate. This improvement is expected because while the cutoff classifier only considers information of co-regulated genes, the SVM classifier includes additional information such as co-regulated genes and co-regulating TFs as well as information about false regulations.

To further evaluate the benefit of knowledge bases for other previously established methods, the NR and KBR were applied to the CLR algorithm [4]. The CLR algorithm estimates a likelihood of the mutual information score between a TF and a TG, and determines the pair to be true or false by comparing the estimated likelihood with its background distribution. In the original CLR algorithm, the mutual information is obtained from gene expression profiles of a TF and a TG, which are the same as their NRs. By substituting NRs to KBRs, the performance improvement gained by incorporating prior knowledge can be measured for the CLR algorithm. As before, KBRs were encoded with the positive training set, and the performance was measured over the test set. While the CLR algorithm with the NRs showed reasonable predictive power for *E. Coli*, it did not predict well for yeast, managing a 15% true positive rate at a 10% false positive rate (Figure 2B). We suspect that the complex regulatory mechanisms of eukaryotic organisms can possibly induce such performance reduction. In contrast, the KBR CLR algorithm showed a much better performance with a 42% true positive rate at the same 10% false positive rate under the same conditions. In addition, the KBR CLR had comparable performance with a 50% true positive rate at 10% false positives of the SEREND (SEmi-supervised REgulatory Network Discoverer) algorithm, which is a supervised approach using a knowledge base that previously showed a significant improvement over the NR CLR for *E. coli *[18]. These results indicate that a knowledge base can help improve the prediction power and accuracy for regulatory relations, and we found that the SVM method utilizing KBR showed over all the best performance (80% true positive rate at 10% false positives).

### Important characteristics of knowledge bases

A knowledge base is a representation of our existing understanding of biology; therefore, it is not complete. Different knowledge bases often vary on the type of original sources-direct vs. indirect evidence of regulation, the comprehensiveness and the quality of the collections. Intuitively, direct, comprehensive and high quality knowledge of regulation relationships are important characteristics of a knowledge base.

To evaluate the directness of a knowledge base, we compared the YPD database [5] and ChIP-chip data [7] with p-value less than 0.0001. The YPD database includes direct knowledge of regulations collected from the literature, while ChIP-chip binding data provides evidence of binding between a TF and a TG. Since the binding of a TF to the DNA of its TG is considered a necessary but not sufficient step in transcriptional activation, ChIP-chip data can be considered as indirect knowledge of transcriptional regulations. We tested these two types of prior knowledge using NR SVM. First, we randomly chose two sets of 2,000 pairs among 3,043 YPD database pairs and 2,789 ChIP-chip binding pairs as positive training sets. Among the rest of the 1,043 YPD database pairs, we chose 996 pairs that are not included in either of the two positive training sets as a positive test set.

**Figure 3:**
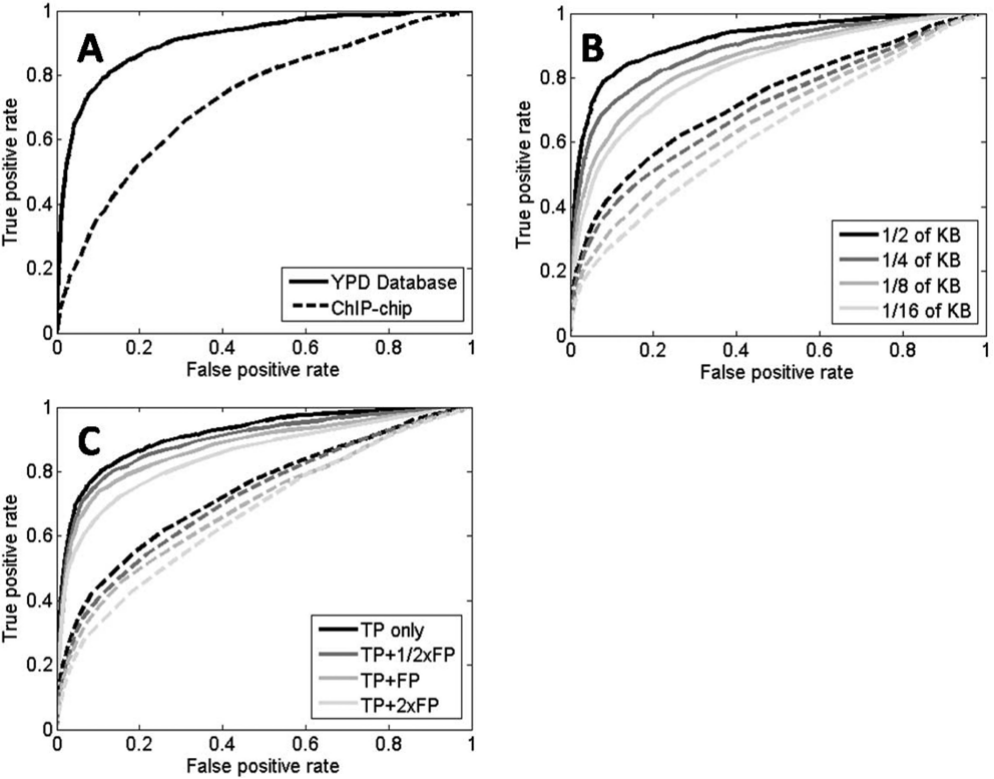
**Performance comparison according to characteristics of knowledge bases**. ROC curves of true and false positive rates for the analysis using **(A) **direct (YPD) and indirect (ChIP-chip) prior knowledge, **(B) **different sizes of knowledge bases with KBR SVM (solid) and KBR cutoff (dashed) methods, and **(C) **different qualities of knowledge bases by adding different amount of false positives to the knowledge base with KBR SVM (solid) and KBR cutoff (dashed) methods. True positive rate is the ratio of samples determined as true from the positive test set, and false positive rate is that from the negative test set

Negative training and test sets were generated according to the distribution of the positive sets. The ROC curves of the two knowledge bases show that the direct knowledge base have better performance than the indirect one (Figure 3A). Another possible explanation for the above observation is that bindings of TFs to DNA were measured in only few conditions, mainly in rich media [7], while the YPD knowledge base has regulatory information collected from vast number of different experiments. Since the microarray expression dataset from Stuart *et al*. [20] was collected from experiments under various conditions, the ChIP-chip results from Harbison *et al*. [7] might not reflect the regulatory relations well across all these experiments.

To evaluate the importance of the size of a knowledge base, we tested KBR cutoff and KBR SVM on several sets of knowledge of different sizes. Each algorithm was applied to build classifiers trained using 1/2, 1/4, 1/8 and 1/16 of the knowledge base, and it was tested on the rest of the knowledge base. As expected, the prediction performance diminished as the size of the prior knowledge decreases (Figure 3B). Since our KBR methods explicitly utilized the averaged expression values of the known TGs to reduce the noises and identify the common signal of regulation, more comprehensive information on the known regulatory relationships lead to the improved sensitivity and accuracy of the predictions.

We analyzed the prediction performance corresponding to different knowledge base qualities by adding different amount of artificial false positive regulatory relations to the existing knowledge base. We built a positive training set with a half of the knowledge base, true positives. We then added random false positives of a half, equal and twice size of the true positives to the positive training set. The other half of knowledge base was used for a positive test set. Negative training and test sets were generated as before. As shown in Figure 3C, the predictions with less false positives in the knowledge base demonstrated better performance for both of KBR SVM and KBR cutoff methods. Since false positives in the training set perturb the predictor and add noise to the averaged expression values that we utilize here, high quality knowledge bases have more advantages in the inference of new regulatory pairs.

### Genome-wide inferences of new regulatory relations

Finally, genome-wide predictions were performed to identify candidate novel regulatory pairs. We used the KBR SVM method that showed the best performance in the cross-validation tests. The full YPD database served as the positive training set, and the negative training set was generated randomly. We tested all possible pairs of 523 TFs and 5,940 genes, and discovered 1,765 candidate regulatory relationship pairs (prediction scores > 0.8). This cutoff threshold corresponds to ~0.01% of false positive rate in the cross-validation tests. Among them, 572 pairs were found to be new regulatory relations not previously included in the YPD database. The re-discovery rate of known regulatory relations was 39.3% (= 1,193/3,043) by selecting top 0.057% (= 1,765/523/5,940) of total candidates.

**Figure 4:**
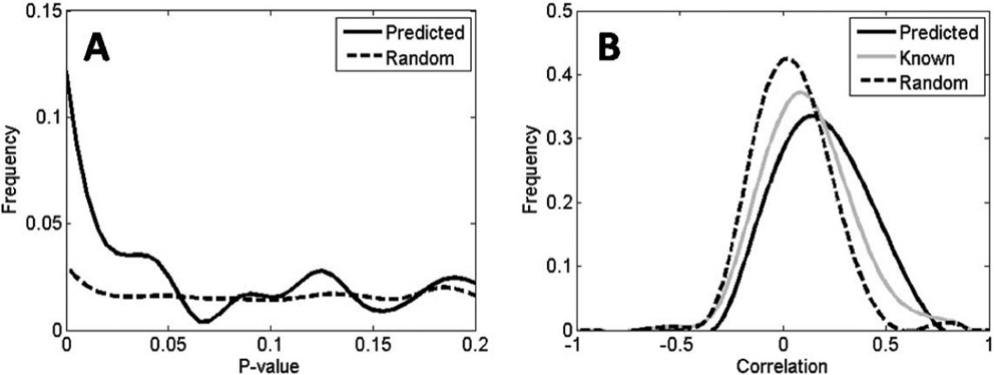
**Validation of the genome-wide regulatory predictions with external resources**. **(A) **P-value distributions of ChIP-chip binding dataset for predicted regulatory pairs and randomly selected pairs. **(B) **Correlation coefficient distributions in the growth fitness dataset for known co-regulated TGs, predicted regulatory pairs and random pairs. The three distributions shown in (B) represent the correlation of growth fitness between a known TG (gray), a predicted TG (solid black), and a randomly selected non-TG vs. the centroid of the known TGs of a TF (dashed black).

While these predicted relations can be verified using individual experiments, the verification experiments are often difficult to conduct at a large scale. We therefore evaluated the predicted regulatory pairs with an independent source of high-throughput data, a well-known ChIP-chip binding dataset [7]. Among 572 predicted pairs, 259 pairs were tested in the ChIP-chip experiment, and these pairs had a significantly lower p-value distribution in binding than randomly selected pairs (Figure 4A). Even though binding of protein to DNA does not guarantee transcriptional activation, most of the transcriptional regulatory processes are expected to include binding. Therefore, lower p-values of the predicted pairs imply that the predicted pairs have higher chance to be verified as true regulatory relations. 12 predicted pairs with prediction scores larger than 0.8 and ChIP-chip binding p-values less than 0.01 are listed in Table 1. Some of the predicted regulatory pairs are indirectly supported by independent studies. For example, the binding of IFH1 to the promoter position of RPS22A was observed independently through high-resolution ChIP [24], and GCN4 was reported to contribute to the induction of ADE12 from microarray experiments on wild-type and GNM4 mutant strains [25]. We also noticed that not all the 259 pairs of candidates had significant p-values in the ChIP experiment, and we suspect that this is partially because the predictions are based on gene expression data measured under many different conditions together with accumulated previous knowledge while the ChIP-chip experiment used for comparison was mainly under rich media. In addition, using the growth fitness dataset of homozygous yeast gene deletions under chemical genomic profiling [19], we examined the phenotypic correlations of co-regulated TGs by the same TFs. As shown in Figure 4B, the co-regulated genes had higher correlation with each other in phenotypes compared with random pairs. We suspect that the phenotypes of deletions of co-regulated TGs tend to have higher correlations because co-regulated TGs tend to have enriched similarity in biological functions. To test this hypothesis for the 572 predicted pairs, we measured the correlation between the fitness of a predicted TG and the fitness centroid of the known TGs of its predicted TF, and compared the distribution with randomly selected pairs. The predicted pairs had significantly higher correlation than the random pairs as previously known pairs (Figure 4B). Rigorous determination controlled by 0.01% of false positive rate can induce higher correlation of the predicted pairs than known pairs. The above tests on these independent experiment datasets suggest that the predicted regulatory pairs can potentially be reasonable candidates for follow-up biological verifications.

**Table 1:** Genome-widely predicted regulatory pairs of TFs and TGs. Listed are predicted pairs of which prediction scores are larger than 0.8 and p-values of binding are less than 0.01.

TFs	TGs	Scores	P-values
YLR223C (IFH1)	YJL190C (RPS22A)	1.2911	0.0070
YJR060W (CBF1)	YOR375C (GDH1)	1.2126	0.0001
YLR451W (LEU3)	YOR202W (HIS3)	1.2027	< 0.0001
YDR207C (UME6)	YJR048W (CYC1)	0.9489	0.0059
YEL009C (GCN4)	YNL220W (ADE12)	0.9472	0.0077
YLR223C (IFH1)	YMR242C (RPL20A)	0.9341	0.0079
YLR403W (SFP1)	YBR189W (RPS9B)	0.8802	0.0050
YDR253C (MET32)	YPR167C (MET16)	0.8625	< 0.0001
YBR083W (TEC1)	YGR032W (GSC2)	0.8542	0.0095
YDL056W (MBP1)	YIR019C (MUC1)	0.8524	0.0006
YLR403W (SFP1)	YGL076C (RPL7A)	0.8429	0.0058
YGL035C (MIG1)	YGR088W (CTT1)	0.8401	0.0091

## Conclusion

In this work, we show that a knowledge-based approach significantly helps the characterization of true gene transcriptional regulatory interactions from non-regulatory random pairs. The expression patterns between co-regulated TGs were much more highly correlated compared to random pairs. In addition, predictions of transcriptional regulations using a knowledge-based approach achieved significantly better performance than using the high throughput genomic data alone. We also examined the impact of several properties of the knowledge base on prediction performance, and showed that the sensitivity and specificity increase when using a high-quality, comprehensive knowledge base with direct regulatory information. Finally, we performed genome-wide prediction over all possible TF-TG pairs and determined 572 pairs as new candidate regulatory relations. These predicted regulatory pairs have significantly lower p-values in ChIP-chip binding dataset and higher correlation in growth fitness with previously known TGs. Overall the results suggest that knowledge-driven analysis significantly helps the interpretation of high-throughput genomic data. Several efforts are underway to build high quality, comprehensive knowledge bases from peer-reviewed publications that integrate genomic, chemical and systemic functional information, including BIND, Proteome, IPKB, KEGG, Reactome and others. These databases will enable further development of statistical and machine learning approaches for the integrative analysis of high-throughput experimental data, especially in the studies of higher organisms. We will further evaluate applying our methods to these studies.

## Competing interests

The authors declare that they have no competing interests.

## Authors' contributions

JS, RD and WX conceived the project and design. JS implemented the algorithms and performed the computational analysis. JS and WX wrote the paper, and AK critically revised it. All authors read and approved the document.
